# Determination of Caffeoylquinic Acids Content by UHPLC in *Scolymus hispanicus* Extracts Obtained through Ultrasound-Assisted Extraction

**DOI:** 10.3390/plants12122340

**Published:** 2023-06-16

**Authors:** Antonio Ruano-González, Ana A. Pinto, Nuria Chinchilla, Miguel Palma, Gerardo F. Barbero, Ceferino Carrera, Mercedes Vázquez-Espinosa

**Affiliations:** 1 Department of Organic Chemistry, Faculty of Sciences, University of Cadiz, 11510 Puerto Real, Spain; antonio.ruano@uca.es; 2 Department of Analytical Chemistry, Faculty of Sciences, Agrifood Campus of International Excellence (ceiA3), Wine and Agrifood Research Institute (IVAGRO), University of Cadiz, 11510 Puerto Real, Spain; miguel.palma@uca.es (M.P.); gerardo.fernandez@uca.es (G.F.B.); ceferino.carrera@uca.es (C.C.); mercedes.vazquez@uca.es (M.V.-E.); 3 Department of Organic Chemistry, Faculty of Sciences, Institute of Biomolecules (INBIO), University of Cadiz, 11510 Puerto Real, Spain; nuria.chinchilla@uca.es

**Keywords:** *Scolymus hispanicus*, caffeoylquinic acids, ultrasound-assisted extraction, antioxidant properties, phenolic compounds, golden thistle

## Abstract

*Scolymus hispanicus* L., also known as golden thistle, Spanish oyster thistle or, more commonly, as tagarnina is a plant that belongs to the Asteraceae family. It is collected from the wild for human consumption in Mediterranean countries. It is a relevant ingredient in Andalusian culinary culture, where the midribs of young plants are harvested for consumption. *Scolymus hispanicus* L. contains a wide variety of phenolic compounds such as caffeoylquinic acids (CQAs), among others. In the present work, the major phenolic compounds present in tagarnina have been identified, with 5-caffeoylquinic acid (5-CQA) and 3,5-dicaffeoylquinic acid (3,5-diCQA) being the main ones. A method based on ultrasound-assisted extraction (UAE) has been developed for the extraction of these compounds, with the percentage of methanol, sample-to-solvent ratio and the pH being the most influential factors. The developed method has been validated and employed to determine the concentration of 5-CQA and 3,5-diCQA in the midribs of *Scolymus hispanicus*, collected in six different places in the south of Spain. The antioxidant activity of the samples has also been determined, and a direct correlation with their caffeoylquinic compounds content has been established, showing an antioxidant effect.

## 1. Introduction

The *Scolymus* genus belongs to the Astaraceae family and is included in the Lactuceae tribe (also known as the Cichorieae tribe). Even though around twenty species have already been described, only four of them have been formally accepted up to present. These plants were traditionally found throughout Mediterranean and Macaronesian regions, but they have also pioneered other regions, such as tropical Africa, North America, South America and Australia [1]. *Scolymus hispanicus* L., *Scolymus maculatus* L., *Scolymus giganteus* Roem. and *Scolymus grandiflorus* Desf. are the species currently accepted to be members of this genus.

The plants of the genus *Scolymus* are mostly used as food. Specifically, *Scolymus hispanicus*, also known as tagarnina, common golden thistle or Spanish oyster thistle, is used as an ingredient in a variety of traditional dishes from Spain, Morocco and other Mediterranean countries [2]. Recent studies have confirmed that it contains high levels of certain minerals, such as calcium, potassium and magnesium, as well as polyphenolic compounds probably related to its antioxidant properties [3]. In Spain in particular, it is mostly consumed in its southern regions as one of the main vegetables in the traditional gastronomy of the inland territories in Cadiz province, where its midribs are harvested and consumed while in their early stages of development (3–4 weeks before the plants’ flowering). Leaves and roots are discarded, while its midrib is chopped to be first boiled and then seasoned with spices and garlic and finally fried. It is also commonly included, together with other vegetables, in some traditional dishes, such as stew. Although these plants have exhibited an extensive range of properties, like many other species from the Mediterranean basin, many of them are still to be described [4,5,6]. It is worth highlighting the antioxidant, antidiabetic and diuretic activity of the extracts obtained from the roots, stems, leaves and flowers of *Scolymus hispanicus*. This activity is associated to the following major compounds found in these fractions: sinapic acid, caffeic acid, gallic acid and chlorogenic acid [5,7,8,9]. In *Scolymus grandiflora*, in particular, the properties of essential oils stand out, in which there are compounds with antiangiogenic properties (inhibit the formation of new blood vessels) used in cancer treatments [10]. Triterpenes have also been isolated from *Scolymus* genus specimens, where taraxasterol acetate is one of the main examples, as a compound found in the root of *Scolymus hispanicus* that displays antispasmodic activity [11]. The major compounds in the plants of the *Scolymus* genus are polyphenols, which are probably related to many of its properties. Since the 1990s, flavonoids have been isolated from the stems, leaves and flowers of *Scolymus hispanicus* [12,13,14,15]. Many of these compounds have anticarcinogenic, anti-inflammatory and antiviral properties, among others [16,17], or cell growth-inhibiting substances [18] also with anti-HIV and antioxidant properties [19]. Other derivatives found in this plant are kaempferol (also reported in *Scolymus maculate* [20]), among others [21,22,23,24].

**Figure 1:**
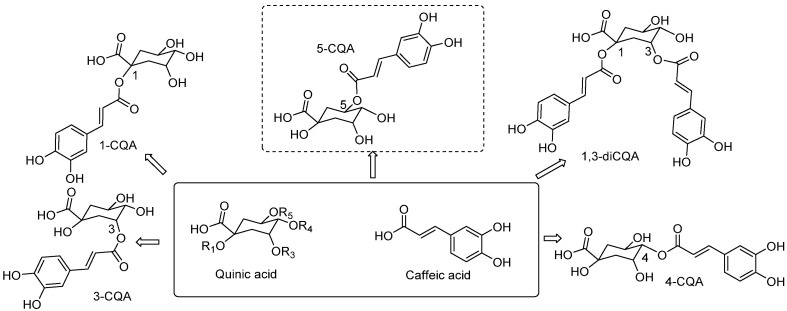
CQAs derivatives from the esterification of quinic acid and caffeic acid.

Caffeoylquinic acids are a family of compounds derived from the esterification of caffeic acid and quinic acid (Figure 1) [25]. These are specialized bioactive metabolites derived from the phenylpropanoid biosynthetic pathway. In plants, CQAs play a defensive role against biotic or abiotic stress [26]. CQAs are phenolic compounds mainly characterized by their interesting biological properties and that have been isolated from a variety of species. It has been proven in recent years that they are important components in the so called “super foods” and that their intake, as a variety of vegetables are incorporated to our diets, is related to certain health promoting activities, such as their antioxidant or anticarcinogenic effects [27,28].

The biological activities associated to CQAs are quite varied. Many studies have linked CQAs consumption to a wide range of properties with health-promoting benefits, including their antioxidant, neuroprotective, antibacterial, antiparasitic, anti-inflammatory, anticarcinogenic, antiviral, antidiabetic and cardiovascular effects, among others [25,29]. In fact, many of these compounds are widely distributed in medicinal plants, which would explain their properties. Preclinical and clinical studies have provided evidence that CQAs might protect against the neurological degeneration resulting from brain oxidative stress, as they play an important role as antioxidants [30,31].

As CQAs act as scavengers of free radicals, their antioxidant activity is one of their most remarkable properties [29]. Thus, CQAs interact with both reactive oxygen species and reactive nitrogen species, donating hydrogen atoms to reactive molecules and transforming them into less active radicals. CQAs also have the beneficial effect of generating reactive oxygen species that stimulate the endogenous cell signals that are required in response to cell injuries while preventing an excessive generation of free radicals within the cell. This is particularly important as some of the important constituents of the cells might be damaged if reactive oxygen species are not properly controlled [32,33,34]. The antioxidant capacity of CQAs has been proven to be related to the number of caffeoyl groups on the quinic acid ring [29]. In fact, the compounds 3,5-diCQA, 4,5-diCQA and 3,4,5-triCQA from the roots of *Arctium lappa* L. (Asteraceae) presented significant free radical scavenging activity according to DPPH, ABTS and FRAP assays [33]. Another example could be the CQAs isolated from *Moquiniastrum floribundum* (Asteraceae), which exhibited anti-radical properties confirmed by DPPH assays [35]. In recent years, there has been a notably growing interest in the study of CQAs for their antiradical activity and their potential ability to effectively treat cognitive and lifestyle-related disorders, among others.

The main objective of the work is the development and optimization of the ultrasound-assisted extraction (UAE) of the major compounds (CQAs) present in *Scolymus hispanicus*. The proposed extraction method (UAE) has been confirmed as one of the most efficient techniques that can be applied to the extraction of metabolites from plant resources. One of UAE’s strong aspects is that it preserves the integrity of the metabolite molecules in the plant over the whole process as it operates at low temperatures. This ensures the successful and complete determination, quantification, and analysis of 5-CQA and 3,5-diCQA, the major compounds present in *Scolymus hispanicus*. This technique is based on the capacity of ultrasounds to break the matrix’s cell walls that conform the plants’ tissues by generating a number of hotspots that give rise to cavitation episodes in the solvent medium. Not only does cavitation increase the transfer of metabolites into the solvent [36,37], but it can also improve the extraction process by modifying the polarity, pH or temperature of the solvent, among other variables [38,39]. This technique has been widely used for the extraction of polyphenols and related compounds from the matrices of different plants. UAE has been confirmed to obtain large extraction yields by using short extraction times (usually less than 10 min), as well as reduced amounts of solvent and plant material (around 10 mL of solvent and less than 0.5 g of sample in function of the compounds to be extracted). For all these reasons, UAE is currently considered as one of the quickest, most economic and environmentally friendly techniques [36,40].

In the present work, the presence of 5-CQA and 3,5-diCQA in *Scolymus hispanicus* midribs has been detected. A UAE method has been developed and optimized using the Box–Behnken design and response surface methodology. For this purpose, the main influential variables on UAE (extraction solvent, temperature, amplitude, pH, sample-to-solvent ratio and cycle) have been studied and optimized for the successful extraction of 5-CQA and 3,5-diCQA from real samples of *Scolymus hispanicus* midribs. The antioxidant activity of each extract has been determined, and a direct correlation between 5-CQA and 3,5-diCQA concentration levels and the antioxidant capacity of the extracts has been confirmed. These results show the potential of *Scolymus hispanicus* as a healthy food, thanks to the antioxidant properties present in its major constituents.

## 2. Results and Discussion 

### 2.1. Solvent Composition Optimization

**Figure 2:**
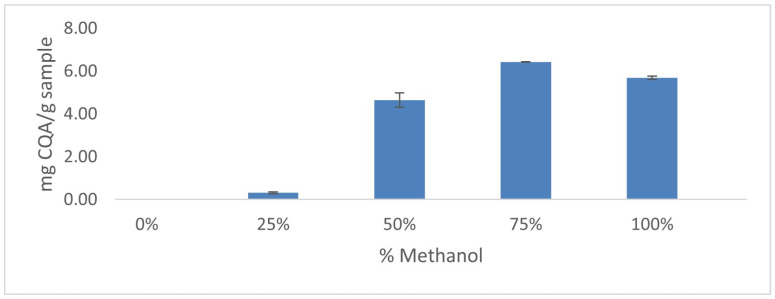
CQA compounds obtained using different percentages of methanol in the extraction solvent (*n* = 3).

In order to determine the optimal percentage of methanol to be used in the solvent, 0.3 g of the mixture of the six lyophilized samples was subjected to triplicate extractions using Milli-Q water:methanol mixtures. For all these extractions, the temperature was set at 40 °C, with an ultrasound amplitude of 60%, a volume of 15 mL solvent and a time of 10 min. These initial conditions had been previously verified, i.e., the extraction of 5-CQA and 3,5-diCQA occurred. The results are shown in Figure 2. It can be observed that a 75% percentage of methanol in the solvent achieved the best results. On the other hand, a high percentage of water (0% and 25% methanol) resulted in minimal amounts of 5-CQA and 3,5-diCQA extracted because of the high polarity of water. Therefore, solvent mixtures containing higher percentages of methanol (50%, 75% and 100%) were selected to carry out the Box–Behnken design (BBD). 

### 2.2. Temperature Stability Study

**Figure 3:**
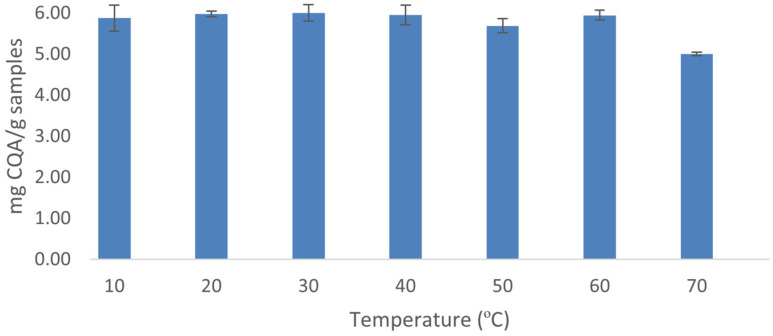
CQA compounds obtained at different temperatures (*n* = 3).

The stability of the CQA compounds at different temperatures was evaluated in a range from 10 °C to 70 °C. The assay conditions were 75% methanol solvent, 60% ultrasound amplitude, 15 mL volume and 10 min time. The extractions have been carried out in triplicate. The total amount of 5-CQA and 3,5-diCQA has been determined at different extraction temperatures to verify the stability of these compounds in ultrasound-assisted extraction. According to the results obtained, the concentration of the compounds of interest remained constant between 10 and 60 °C (Figure 3). Since temperature did not affect the amount of CQA compounds obtained at this range, it could be concluded that temperature did not have any effect with regard to their degradation. Therefore, a range of temperatures going from 10 °C up to 60 °C was selected for the optimization of the extraction method.

### 2.3. UAE Method Optimization

The efficiency of UAE for the extraction of the major phenolic compounds that are present in *Scolymus hispanicus* was determined for its optimization. A Box–Behnken design with six variables (solvent methanol percentage, temperature, sample-to-solvent ratio (ratio), pH, ultrasound amplitude and cycle) was employed to determine the optimal conditions for the extraction of the main phenolic compounds (5-CQA and 3,5-diCQA). The parameters’ ranges used are shown in Table 1.

For the optimization process, 54 experiments were carried out. All of them were set up at 10 min extraction time. The statistical application Statgraphic Centurion (Version XVIII) was used for the analysis of the data as well as for the identification of the most influential parameters, quadratic factors and interactions (*p* ˂ 0.05) with regard to optimal operating conditions. The analysis of the results (ANOVA) allowed to recognize the most relevant factors and their possible interactions. The *p*-values and estimated coefficients for the different parameters in the quadratic equation generated according to the design are shown in Table 2. It can be observed from these results that methanol percentage (A), ratio (C), pH (D) and the double interaction %MeOH-%MeOH (AA) were the most influential parameters at 95% confidence level, as variables with a *p*-value lower than 0.05 were considered as influential.

**Figure 4:**
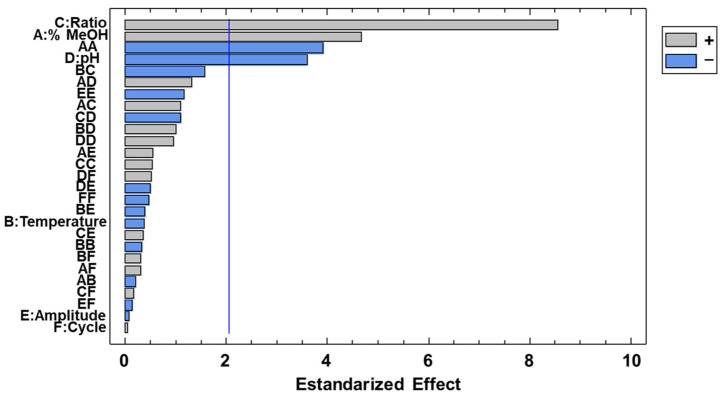
Pareto chart showing the effect of the studied variables and their interactions on the response variable, i.e., total CQAs.

A Pareto chart (Figure 4) allowed to detect the most significant parameters and their interactions at 95% significance level. Thus, those values that exceeded 2.17 were the ones to be considered as the most relevant. It can therefore be observed that sample-to-solvent ratio, %MeOH, %MeOH-%MeOH and pH were the most influential variables. Furthermore, the Pareto chart provided additional information with respect to the direct or inverse correlations existing between each factor and the response variable. 

Based on these results, a second order polynomial equation that would allow to correlate the response variable with the most influential factors and interactions has to be generated. The factors and interaction with relevant regression coefficients, i.e., those presenting a *p*-value lower than 0.05, were considered to have a significant effect and, therefore, should be included in the equation. Thus, according to the data in Table 2, Equation (1) was generated as follows to determine total caffeoylquinic acids (TCQAs):

(1) $$\mathrm{TCQAs}=-1.27214E6+44703.2\;x\;\%\mathrm{MeOH}+2.27012E6\;x\;\mathrm{Ratio}-254745\;x\;\mathrm{pH}-357.327{x\;\%\mathrm{MeOH}}^{2}$$

**Figure 5:**
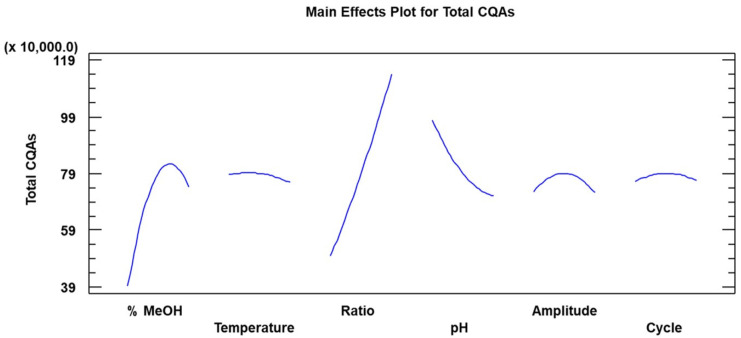
CQAs extraction trends obtained by applying Equation (1) using a range of increasing values for each variable parameter.

This polynomial equation allowed us to determine the trend followed by each factor. Therefore, it was applied to each factor in the model by assigning each one of them a specific range of increasing values while the rest of the parameters remained constant at a mid-level value. By plotting the trends corresponding to each parameter according to Equation (1) (Figure 5), the variations displayed by the response variable that could be attributed to each parameter were identified. 

Based on the results displayed by the most influential factors (% methanol, ratio and pH), the following conclusions were reached: the extraction of phenolic compounds improved as methanol percentage was increased up to a point where the response variable started to fall. It can be deduced that the compounds of interest are not extremely polar and that, therefore, greater extraction was obtained as the percentage of methanol increased until a maximum of 81%. At this point, an excessive polarity inhibited the extraction of the phenolic compounds [25,29]. 

The increasing value of the sample ratio resulted in a linear trend where the extractions were greater as the sample ratio went up. This explains why the optimum ratio was reached at 0.3 g sample. We should assume that if we had increased this ratio above 0.3 g, greater extraction values would have been obtained as the solution would have been more saturated, while when smaller amounts of samples were used, it would have been more diluted. However, sample amounts greater than 0.3 g generated extracts that were difficult to process, and the signal obtained in the chromatograms was not adequate (excessive noise), so a ratio of 0.3 g/20 mL of solvent was established as optimal for the extraction.

The variations in pH levels triggered protonation or deprotonation reactions in the feed, which caused changes in its charge and in its conjugated double bonds, which resulted in different structures of modified color and, in some cases, unstable compounds [41,42,43,44]. Finally, the statistical analysis of the results allowed to determine the optimal operating conditions for the extraction of 5-caffeoylquinic and 3,5-dicaffeoylquinic acid by UAE (Table 3).

Regarding the optimum percentage of methanol to extract the CQAs from *Scolymus hispanicus*, 81% MeOH in water has been obtained as optimum. At this proportion of solvents, the solvation of the CQAs will be maximum, corresponding to polarities equivalent to that of the compounds under study [45,46,47].

The temperature, amplitude and cycle presented similar behaviors, with intermediate values being the optimal ones so that pH was established at 3—just one point above the lower limit (pH = 2)—unlike the sample ratio, which was positioned at the upper end of the range. 

### 2.4. Optimal Extraction Time

**Figure 6:**
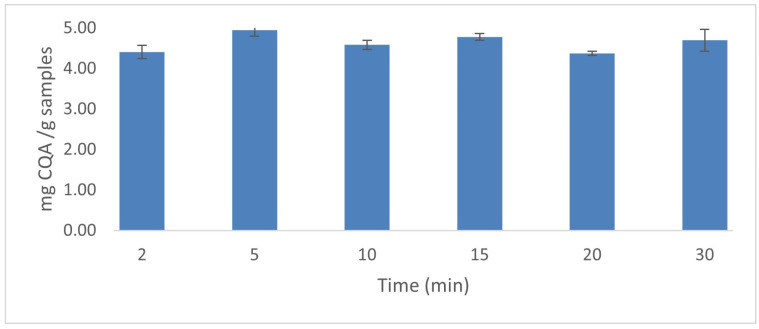
CQAs yields obtained using different extraction times under the established optimal conditions (*n* = 3).

Once the optimal conditions for the extraction of major phenolic compounds by UAE were established, several experiments, under such conditions, were carried out in triplicate and using times from 2 to 30 min to determine the best extraction time. The data from these experiments showed that the greatest yield was obtained at 5 min (Figure 6) and that when using longer times, less amount of CQAs are extracted.

### 2.5. Repeatability and Intermediate Precision of the Developed Method

Finally, for the validation of the method, repeatability and intermediate precision studies were carried out. Thus, in order to evaluate its intermediate precision, 18 experiments were completed over three days, while 9 experiments were completed on the same day in order to determine its repeatability. The results have been included in Table 4.

Since 5% coefficient of variation is the standard reference value for this type of tests, we can conclude that the developed method presented good repeatability and intermediate precision.

### 2.6. Application to Real Samples 

The optimized method was applied to different samples of *Scolymus hispanicus,* collected from six different areas nearby Paterna de Rivera (Spain) on April 26th, 2022. The roots and leaves were removed and discarded for the determination of 5-CQA and 3,5-diCQA in the midribs, which is the part of the plant normally used as a gastronomic resource. The optimized extraction method according to the Box–Behnken design was applied to all the plant samples. Table 5 shows the locations and soil moisture content of the six sampled areas.

The collected areas presented different humidity levels, with Zone 5 (La Joya) containing the highest one. Zones 2, 3 and 6 presented some soil moisture, although at a lower level than Zone 5. Finally, Zones 1 and 4 were quite dry and had little water availability for the plants. 

The analysis of the concentrations of 5-CQA and 3,5-diCQA (Table 6) in these plants suggested a notable correlation with the state of soil moisture. It is observed that the highest concentration of CQAs (6.74 mg/g sample) corresponds to the sample from Zone 5 (higher amount of soil moisture). The samples from Zones 2, 3 and 6 (also with available moisture in the soil, although with less abundance than Zone 5) also present quite high values of CQAs (5.38; 4.85; 4.38 mg/g sample, respectively) although lower to those found in Zone 5. Finally, it is observed that those samples collected in areas with very little water availability (Zone 1 and 4) present much smaller values of CQAs (1.79; 1.66 mg/g sample, respectively) than areas where there is some availability of water. It is therefore observed that there is a marked relationship between the level of water available to the plant and the amount of CQAs produced by it. Similar results were obtained by Pandino et al. in *Cynara cardunculus* L., where they observed that the production of CQAs is 28% higher in plants with 100% water supply compared to plants with 75% [48].

### 2.7. Antioxidant Capacity of the Real Samples

All the samples were analyzed by DPPH. The results from these analyses have been presented in Table 5. The samples from “La Joya” (Zone 5) and “Las Piletas” (Zone 2) displayed the highest antioxidant activity, while the samples from “El Salao” (Zone 1) and “La Peña” (Zone 4) exhibited the lowest ones, as the plants from both of the latter spots presented lower concentrations of 5-CQA and 3,5-diCQA. These results confirm how important the growth stage of the plants is with regard to the antioxidant capacity of their extracts. It should be mentioned that the antioxidant activity values registered for the plants from “La Joya” (Zone 5) are comparable to those of artichoke (*Cynara scolymus*), of the Blanc d’Oran variety, which is around 19 mg trolox equivalent/g sample [49]. It should be finally noted that the extracts’ antioxidant capacity was closely correlated with the presence of 5-CQA but specially related with the higher concentration of 3,5-diCQA (Table 6). This fact is in agreement with the data reported by Liu et al., where they indicate that the antioxidant capacity of CQAs is related to the number of caffeoyl groups in the quinic acid ring [29].

## 3. Materials and Methods

### 3.1. Biological Samples

*Scolymus hispanicus* midribs have been used for this study. These were collected from several wild spots around Paterna de Rivera (Cadiz, Spain) (Table 5). The harvesting method was the same in every case: the specimens were placed in labeled bags in order to identify their collecting spot and taken to the laboratory to be stored at −20 °C. The plants’ midribs were subjected to a lyophilization process, consisting of freezing the samples at −80 °C (CVF 525/86, Ingeniería de Climas para Procesos, Barcelona, Spain) and then lyophilizing them by means of a VirTis BenchTop K lyophilizer (SP Scientific, Warminster, PA, USA). The ground lyophilized samples were kept in a freezer at −20 °C. A mixture of the 6 different samples collected (same weight of each lyophilized sample) has been used for the development of the extraction method.

### 3.2. Solvents and Reagents

For the ultrasound-assisted extraction, mixtures of methanol (Fischer Chemical, Loughborough, United Kingdom) of HPLC purity and Milli-Q water, obtained by means of a Millipore purification system (Bedford, MA, USA), were used as solvents. For the extractions at different pH (8, 5 and 2), a 1 M HCl solution (Panreac, Barcelona, Spain) and a 1 M NaOH solution (Panreac, Barcelona, Spain) were used. The pH measurements were carried out using a Crison GLP22 pH-meter (Barcelona, Spain).

For the chromatographic separations, the solvents used were 100% (glacial) acetic acid (Merck, Darmstadt, Germany), Milli-Q water obtained through a Millipore water purification system (Bedford, MA, USA), acetonitrile and methanol grade HPLC (Panreac, Barcelona, Spain). 

Chlorogenic acid (Sigma-Aldrich Chemical Co., St. Louis, MO, USA) was the standard selected for the quantification of 5-caffeoylquinic acid, according to the following calibration curve: y = 26,442x + 11,415, with a coefficient of determination R^2^ = 0.9999. For the diesterified derivative 3,5-diCQA, cynarin was the standard used for the quantification according to the following calibration curve: y = 400.56x + 3101.6, with a coefficient of determination R^2^ = 0.9996. Cynarin was isolated and purified by SPE (Strata X 200 mg/3 mL, Phenomenex, Torrance, CA, USA) from a commercial lyophilized extract of *Cynara scolymus* L. (PRIDAHO-FARMA, S.L., León, Spain) and employing 30% MeOH/H_2_O.

### 3.3. Ultrasound Assisted Extraction

The ultrasonic-assisted extractions were carried out using a “Sonopuls Ultrasonic Homogeniser HD4100” probe (Bandelin, Berlin, Germany), coupled to a processor that allows the adjustment of both amplitude and duty cycle. A thermostatic bath (Frigiterm-10, JP Selecta, Abrera, Spain) was also used to adjust the temperature as desired. 

Lyophilized and finely ground *Scolymus hispanicus* midrib 20 mL samples at different methanol/water percentages were carefully weighed and placed inside the extraction vessel. The extractions from real samples were performed under the optimal conditions that had been previously determined. After completing the extraction, the supernatant was separated from the solid material by centrifugation (JP Selecta, Abrera, Spain) at 7500 rpm (9.5 cm orbital radius) for 5 min at room temperature. The precipitate was rinsed using fresh solvent, and the resulting liquid was combined with the supernatant up to the volume indicated by the experiment design. The samples for UHPLC were obtained by filtering the extract through a 0.22 μm nylon syringe filter (Membrane Solutions, Dallas, TX, USA).

#### 3.3.1. Validation of the Extraction Method

For the validation of the method, the accuracy of ultrasound-assisted extraction (UAE) was evaluated. Repeatability was assessed by completing nine extractions on the same day (*n* = 9), while the intermediate precision assessment was based on six daily extractions on three consecutive days (*n* = 6 + 6 + 6). The precision of the method was expressed as the coefficient of variation (%CV) of the responses obtained [50].

#### 3.3.2. Box–Behnken Experiment Design

A Box–Behnken Design (BBD) was employed for the optimization of the extraction process. This type of design is characterized by presenting only three levels per factor: a lower level (−1), an intermediate level (0) and a higher level (1). However, the feature that differentiates this design from others is that in addition to not having an embedded factorial or fractional factorial design, it does not present axial points. Instead, it has a more spherical arrangement of the design points [51].

In this work, a Box–Behnken design was developed based on the six variable parameters considered, i.e., percentage of methanol, temperature, pH, amplitude, cycle and sample-to-solvent ratio, and on the response variable, i.e., the total phenolic compounds content. Thus, the experimental design involved 54 experiments with six central points so that the error could be calculated. A response surface methodology applied to the experimental results allowed to generate the mathematical model that best fits the experimental responses according to the conditions used. Thus, a second order polynomial equation (Equation (2)) was obtained where the response of our system was expressed as a function of the variable parameters and their interactions [51].

(2) $$Y=\beta_{0}+\overset{k}{\underset{i=1}{\sum }}\beta_{i}\times {\;X}_{i}+{\;\beta }_{\mathrm{ii}}\times {\;X}_{i}^{2}+\underset{i}{\sum }\overset{k}{\underset{i=1}{\sum }}\beta_{\mathrm{ij}}\times {\;X}_{i}X_{j}+r$$

In Equation (2) “Y” stands for the response, “βi” for the coefficient of each main effect, “βij” for the coefficient corresponding to the interaction between factor “i” and factor “j”, “βii” for the coefficient of the quadratic factors that represent the curvature of the surface, “x” for each of the parameters considered and “r” for the residual value or random error.

### 3.4. Identification of CQAs by UHPLC-QToF-MS

The amount of phenolic compounds in the samples was determined by means of an ultra-high performance liquid chromatographer coupled to a time-of-flight quadrupole mass spectrometer (UHPLC-QToF-MS, Xevo G2, Waters Corp., Milford, MA, USA). Their mass spectra were acquired in negative ion mode under the following conditions: desolvation gas flow = 700 L h^−1^, desolvation temperature = 500 °C, cone gas flow = 10 L h^−1^, source = 150 °C, capillary voltage = 700 V, cone voltage = 30 V and collision energy = 20 eV. Full scan mode (*m*/*z* = 100–1200) was used. 

A 2.1 × 100 mm reverse-phase C18 analytical column with 1.7 µm particle size (ACQUITY UPLC CSH C18, Waters) was employed. A mobile phase A formed by 2% formic acid–water solution and a mobile phase B containing a 2% formic acid–methanol solution were used at a flow rate of 0.4 mL min^−1^. The time and % solvent B gradient employed were the following: 0.00 min, 15%; 3.30 min, 20%; 3.86 min, 30%; 5.05 min, 40%; 5.35 min, 55%; 5.64 min, 60%; 5.95 min, 95%; and 7.50 min, 95%. The total run time was 12 min, including 8 min for the analysis and 4 additional minutes for re-equilibration. The mass spectra were acquired in negative ion mode under the following conditions: desolvation gas flow = 700 L h^−1^, desolvation temperature = 500 °C, cone gas flow = 10 L h^−1^, source temperature = 150 °C, capillary voltage = 700 V, cone voltage = 30 V and collision energy = 20 eV. The ions were scanned from 100 *m*/*z* to 1200 *m*/*z*.

Prior to their identification, all the UAE extracts were filtered through a 0.20 µm nylon syringe filter (Membrane Solutions, Dallas, TX, USA), and 3 µL was the volume injected. The compounds were individually identified on the basis of their retention time and molecular weight. The following 2 major CQAs were identified: 5-caffeoilquinic acid (*m*/*z* = 353) (Figure S1) and 3,5-dicaffeoilquinic acid (*m*/*z* = 515) (Figure S2).

### 3.5. Detecting CQAs by Ultra High-Performance Liquid Chromatography (UHPLC)

The phenolic compounds present in the *Scolymus hispanicus* samples were detected by means of an ACQUITY UPLC H-Class liquid chromatography system (Waters Corporation, Milford, MA, USA) coupled to an ACQUITY photodiode array detector (PDA). The PDA detector was set to a wavelength range from 240 to 400 nm for 3D scanning using a sampling rate of 40 pts s^−1^. 

An ACQUITY UPLC^®^ BEH C18 (2.1 × 100 mm, particle size 1.7 μm, Waters, Milford, MA, USA) column was used for the separation of the phenolic compounds. The temperature was set at 47 °C, and the mobile phase used was a binary system consisting of Milli-Q water acidified with 2% acetic acid as solvent A and acetonitrile acidified with 2% acetic acid as solvent B. The analyses were performed in 8 min using the following time and %B gradient: 0 min, 0%; 1 min, 0%; 3 min, 5%; 4 min, 10%; 4.5 min, 10%; 5 min, 20%; 7 min, 20%; 8 min, 30%. The flow was set at 0.6 mL/min. The CQAs analyzed have been quantified at a wavelength of 320 nm. Figure S3 corresponds to a typical chromatogram of a *Scolymus hispanicus* extract.

### 3.6. Antioxidant Activity

A number of different techniques can be used to assess the antioxidant activity of foods or plants. Nevertheless, the technique based on the usage of the free radical 2,2-diphenyl-1-picrylhydrazyl, commonly known as DPPH, has recently received special attention. The procedure designed by Brand-Williams et al. [52] and modified by Miliauskas et al. [53] is the one we have used for our study as explained below. 

A 6 × 10^−5^ M DPPH solution in methanol was used. For every 100 μL of tagarnina extract, 2 mL of the DPPH solution was added. The mixture was incubated for 40 min in the absence of light and at room temperature. Then, its absorbance at 515 nm was measured. The results were expressed as mg trolox equivalents (TE) per g sample dry weight. For this purpose, a trolox calibration curve (y = 0.9059x − 3.6649; R^2^ = 0.9993) was generated using six points (0–1.4 mM) in triplicate.

### 3.7. Statistical Analysis

The statistical program Statgraphic Centurion Version XVIII (Statpoint Technologies, Inc., The Plains, VA, USA) was used for the optimization of the UAE process. An analysis of variance (ANOVA, *p* ˂ 0.05) was used to determine the significance of the differences between means. The *p*-values and the coefficients of the different parameters, according to the quadratic equation obtained from the design, were registered.

## 4. Conclusions

An UAE method for the production of extracts containing the major phenolic compounds with biological activity that can be found in tagarninas (*Scolymus hispanicus* L.) has been developed and optimized. The UAE method developed has been confirmed suitable for the successful extraction of the said phenolic compounds. For the development and optimization of the extraction method, the following parameters were considered: percentage of methanol in the extraction solvent, temperature, pH, sample-to-solvent ratio and time. The final optimized method presented a high repeatability and intermediate precision, with coefficients of variation lower than 5%. The developed method was applied to midrib samples of *Scolymus hispanicus* L. specimens collected from different spots around Paterna de Rivera (Spain). The amounts of 5-caffeoylquinic acid and 3,5-dicaffeoylquinic acid in the final extracts from the midribs of *Scolymus hispanicus* L. were quantitatively determined. Thus, 5-caffeoylquinic acid was determined as the major compound of interest found in *Scolymus hispanicus*, while 3,5-dicaffeoylquinic acid was confirmed as the second most abundant phenolic compound. The antioxidant activity of the extracts obtained was determined, and a direct correlation between this and the amount of caffeoylquinic acids present in the samples was corroborated.

## Figures and Tables

**Table 1:** Ultrasound-assisted extraction variables and ranges used for the optimization of the extraction method through a Box–Behnken design.

Factor	Low Level (−1)	Intermediate Level (0)	High Level (+1)
% MeOH	50	75	100
T (°C)	10	35	60
Ratio (g)	0.1	0.2	0.3
pH	2	5	8
Amplitude (%)	20	40	60
Cycle (s)	0.2	0.6	1.0

**Table 2:** ANOVA of the response variable values.

Variable	*p*-Value	Estimated Coefficient	Variable	*p*-Value	Estimated Coefficient
A: % MeOH	0.0001	−1.27214 × 10^6^	BD	0.3236	−34,003.5
B: T (°C)	0.7148	44,703.2	BE	0.6983	1086.55
C: Ratio (g)	0.0000	4256.03	BF	0.7553	−29.9245
D: pH	0.0013	2.27012 × 10^6^	CC	0.5985	2267.5
E: Amplitude (%)	0.9437	−254,745	CD	0.2824	3.04617 × 10^6^
F: Cycle (s)	0.9709	14,799.6	CE	0.7223	−355,591
AA	0.0006	−248,702	CF	0.8609	11,635.9
AB	0.8393	−357.327	DD	0.3466	270,292
AC	0.2821	−17.6942	DE	0.6229	13,694.2
AD	0.1999	28,468.3	DF	0.6062	−806.016
AE	0.5861	1205.1	EE	0.2549	56,353.1
AF	0.7640	71.4463	EF	0.8892	−166.323
BB	0.7465	2621.08	FF	0.6473	−151,854
BC	0.1275	−20.7393	Constant	-	−1.27214 × 10^6^

**Table 3:** Optimal values determined by means of a Box–Behnken design.

Factor	Optimal Levels
% MeOH	81
T (°C)	40
Ratio (g)	0.3
pH	3
Amplitude (%)	52
Cycle (s)	0.6

**Table 4:** Precision and repeatability tests of the developed method.

	Repeatability	Intermediate Precision
Media (mg/g)	5.79	5.67
Standard deviation (mg/g)	0.19	0.26
Variance coefficient (%)	3.36	4.61

**Table 5:** Location of the spots from which the specimens of *Scolymus hispanicus* were collected.

Zone	Name	Latitude	Longitude	Altitude	Soil Moisture
1	El Salao	36°31′45″ N	5°54′03″ W	57 m	+
2	Las Piletas	36°31′43″ N	5°57′05″ W	93 m	++
3	Pozo la Lapa	36°32′18″ N	5°53′14″ W	70 m	++
4	La Peña	36°30′46″ N	5°49′29″ W	100 m	+
5	La Joya	36°29′57″ N	5°46′10″ W	81 m	+++
6	Magaña	36°30′45″ N	5°43′49″ W	108 m	++

+ (It corresponds to a dry soil with low hydration at the time of sample collection); ++ (It corresponds to a soil with average hydration at the time of sample collection); +++ (It corresponds to a soil with good hydration at the time of sample collection).

**Table 6:** Antioxidant capacity (mg trolox equivalent/g sample) and individual and total content of CQAs (mg CQA/g sample) of the extracts from *Scolymus hispanicus* midribs.

	Zone 1: El Salao	Zone 2: Las Piletas	Zone 3: Pozo la Lapa	Zone 4: La Peña	Zone 5: La Joya	Zone 6: Magaña
mg trolox equivalent/ g sample	5.39 ± 0.11	11.66 ± 0.21	9.79 ± 0.31	4.54 ± 0.12	17.41 ± 0.21	9.17 ± 0.22
mg 5-CQA/g sample	1.36 ± 0.07	4.30 ± 0.05	3.31 ± 0.20	1.24 ± 0.09	4.15 ± 0.10	2.82 ± 0.12
mg 3,5-diCQA/g sample	0.43 ± 0.04	1.08 ± 0.08	1.54 ± 0.03	0.42 ± 0.05	2.59 ± 0.06	1.56 ± 0.06
mg total CQA/g sample	1.79 ± 0.06	5.38 ± 0.07	4.85 ± 0.12	1.66 ± 0.07	6.74 ± 0.08	4.38 ± 0.09

## Data Availability

The data presented in this study are contained within the article.

## Supplementary Materials

The following supporting information can be downloaded at: https://www.mdpi.com/article/10.3390/plants12122340/s1, Figure S1: Mass spectrum obtained for 5-CQA by UHPLC-Q-ToF-MS; Figure S2: Mass spectrum obtained for 3,5-diCQA by UHPLC-Q-ToF-MS; Figure S3: Chromatogram obtained by UHPLC-DAD from extracts of *Scolymus hispanicus* (λ = 320 nm); (1: 5-CQA; 2: 3,5-diCQA).
