# Supplementary material for: Determination of Caffeoylquinic Acids Content by UHPLC in Scolymus hispanicus Extracts Obtained through Ultrasound-Assisted Extraction
Source: Plants (Basel). 2023 Jun 16;12(12):2340. doi: 10.3390/plants12122340 (PMC10302805; doi:10.3390/plants12122340)
Supplement: Supplementary file 1 [file plants-12-02340-s001.zip › plants-2401295-supplementary.pdf]

Determination of Caffeoylquinic Acids Content by UHPLC in Scolymus hispanicus Extracts Obtained through Ultrasound Assisted Extraction

Antonio Ruano-González [1], Ana A. Pinto [1,2,*], Nuria Chinchilla [3], Miguel Palma [2], Gerardo F. Barbero [2], Ceferino Carrera [2] and Mercedes Vázquez-Espinosa [2]

Supplementary Material

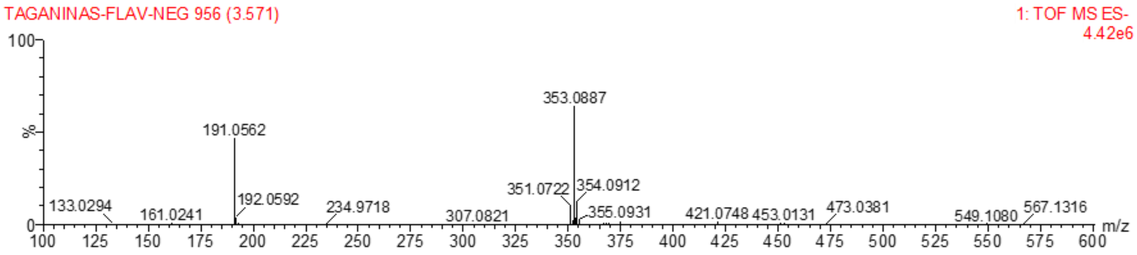

Figure S1. Mass spectrum obtained for 5-CQA by UHPLC-Q-ToF-MS.

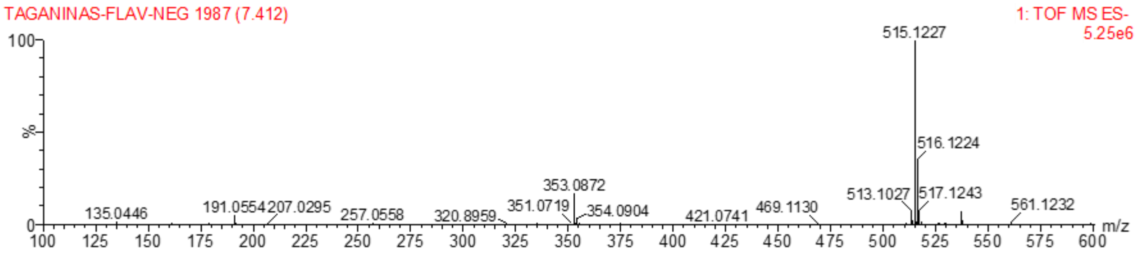

Figure S2. Mass spectrum obtained for 3,5-diCQA by UHPLC-Q-ToF-MS.

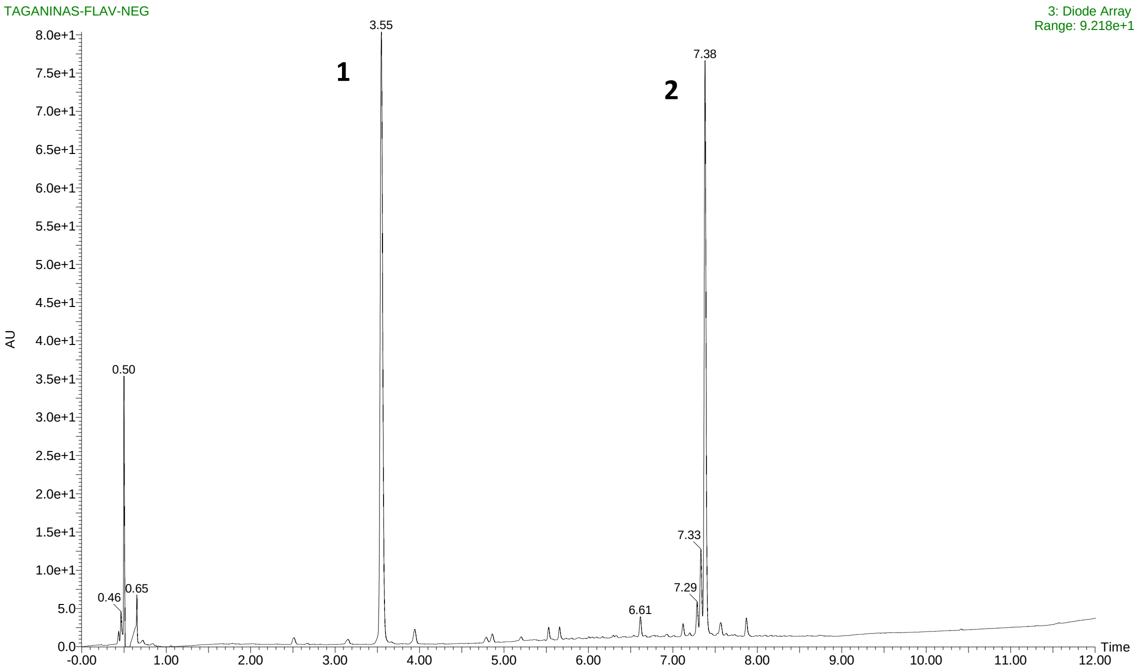

Figure S3. Chromatogram obtained by UHPLC-DAD from extracts of *Scolymus hispanicus* (λ = 320 nm); (1: 5-CQA; 2: 3,5-diCQA).
